# A Role for Estrogen Receptor alpha36 in Cancer Progression

**DOI:** 10.3389/fendo.2020.00506

**Published:** 2020-07-31

**Authors:** Maria Teresa Pagano, Elena Ortona, Maria Luisa Dupuis

**Affiliations:** Center for Gender Specific Medicine, Istituto Superiore di Sanità, Rome, Italy

**Keywords:** estrogen, estrogen receptors, estrogen receptor 36, signaling, cancer, breast cancer, proliferation, autoantibodies

## Abstract

Estrogen receptor α (ERα) functions as a ligand dependent transcription factor that directly binds specific estrogen responsive elements, thus regulating the transcription of estrogen sensitive genes. ERα has also been shown to be associated with the plasma membrane (membrane associated ERα, mERα), concentrated in lipid rafts, plasma membrane microdomains with a distinct lipid composition, where it transduces membrane-initiated estrogen-dependent activation of the mitogen-activated protein (MAP) kinase signaling pathway. Two isoforms of ERα have been described: the “traditional” ERα66 (66 kDa) and a lower molecular weight variant: the ERα46 (46 kDa). More recently, a novel ERα variant with a molecular mass of 36 kDa (ERα36) has been discovered. Notably, ERα36 has been found expressed in different human tumor cells, including both ER- positive and ER- negative breast cancer cells. Estrogen signaling at the cell membrane via ERα36 appears as capable of activating multiple pathways of importance for cancer aggressiveness and metastatic potential. The presence of serum autoantibodies reacting with mERα (anti-ERα Abs) in a large percentage of patients with breast cancer has recently been reported by our group. These anti-ERα Abs seem to act as estrogen agonists rapidly triggering MAP kinase pathway activation thus inducing tumor cell proliferation and overcoming cell resistance to anti-estrogen drug tamoxifen. In this review, we describe the involvement of ERα36 in different tumors. We also report the potential pathogenetic activity of anti-ERα Abs and their implication in drug resistance.

## Introduction

The biological effects of estrogen are mediated by specific receptors designated as estrogen receptors (ERs) (1). In humans, ERs play a key role in reproductive processes and are involved in the regulation of many physiological processes in several tissues, organs and systems such as central nervous system, cardiovascular and immune system. ERs belong to the steroid hormone superfamily of nuclear receptors, i.e., mainly detectable in the cell nucleus. However, ERs have also been found in the cytoplasm and even at mitochondrial level. Two different types of ERs have been identified: the estrogen receptor alpha (ERα) (2, 3), and the estrogen receptors beta (ERβ) (4, 5). Both ERα and ERβ are composed of several functional domains that serve specific roles (6, 7). Starting from NH_2_- to COO-terminus, the principal domains are (i) the N-terminal A/B domain (NTD); (ii) the DNA-binding domain (DBD); (iii) the ligand-binding domain (LBD). Two activation function (AF) domains, AF1 and AF2, located within the NTD and LBD, respectively, appear as responsible for regulating the transcriptional activity of ER. The regulatory mechanisms exerted by estrogens are mainly carried out *via* the control of gene transcription. This can occur after activation and dimerization of ERs, by binding an estrogen response element (ERE, AGGTCAnnnTGACCT). However, estrogen can also directly bind DNA through the involvement of cytoplasmic signaling proteins (8) and growth factor receptors (9, 10). Moreover, further estrogen signaling pathways have been observed. In fact, ERs can also be detected at the plasma membrane (membrane associated ER, mER), where they are embedded in lipid rafts, cholesterol enriched plasma membrane microdomains with a distinct lipid composition. These ERs can ignite non genomic pathways such as the activation of the mitogen-activated protein (MAP) kinase signaling pathway (11, 12). In the same vein, more recently, a seven-transmembrane receptor G protein-coupled receptor 30 (GPR30) structurally unrelated to the other ERs but able to mediate rapid non-genomic signals has also been identified (13).

In humans, the ERβ is 530 amino acids in length, with a molecular weight of 59 kDa and it is encoded by the gene estrogen receptor 2 (ESR2), located on chromosome 14, locus 14q23.2 (14). To date, three other truncated shorter isoforms at 54, 49, and 44 kDa and one elongated isoform at 61 kDa are known. The ERα is encoded by the ESR1 gene located on chromosome 6, locus 6q25.1 (15). The full-length size of ERα is 595 amino acids with a molecular weight of 66 kDa (ERα66). In the last few years, two further shorter isoforms (at 46 and 36 kDa) have been characterized. ERα46, the 46 kDa isoform of ERα, lacking of the N-terminal A/B or of the transcriptional activation domain 1 (AF-1), is expressed in various cell types, as macrophages (16), vascular endothelial cells (17), osteoblasts (18) and also in cancer cells. The other isoform, ERα36, the 36 kDa isoform of ERα, differs from the classic ERα66 in the lack of the AF-1 and AF-2 transcriptional activation domains but it retains the DNA-binding domain as well as the partial dimerization and ligand-binding domains (19). ERα36 shares a common overall structure with ERα46 but it is characterized by a unique 27 amino acids domain that replaces the last 138 amino acids encoded by both ERα46 and ERα66 gene. This unique amino acid sequence in ER36 may alter the ligand binding domain, which explains why ER36 has a different binding affinity. This receptor is mainly located in cytoplasm and at the plasma membrane of several different cancer cell types (19–21) and even in healthy tissues, among which ovarian, breast, kidney, lung, heart and bone (22).

## Estrogen Receptor Alpha 36 Molecular Mechanisms

ERα36 is mainly involved in the initiation of non-genomic signaling pathways to activate the phosphatidylinositol-3-kinase/AKT (PI3K/AKT) and the mitogen-activated protein kinase/extracellular signal-regulated kinase (MAPK/ERK) (19, 23). The interaction of ERα36 with 17β-estradiol (E2) causes Src activation inducing downstream cascades: MEK activation, phosphorylation of ERK and paxillin (PXN), which induces a third messenger expression, cyclin D1 (24). ERα36 also activates ERK1/2 through the protein kinase C (PKC) delta signaling pathway, and phospholipase D (PLD), leading to an increase in the expression of cyclin D1/cyclin-dependent kinase 4, which regulates cell cycle progression, leading to an increase in the proliferative rate and to an enhancement of metastatic potential (25). Through ERα36, E2 and tamoxifen induce the activation of MAPK/ERK and PI3K/AKT pathways that, in turn, regulate c-Myc protein expression, contributing to the cancer metastatic potential (26, 27). More in general, several studies suggest that ERα36 could act as a negative dominant regulator of estrogen genomic signaling promoted by ERα66 and ERβ (28). However, there is a positive feedback mechanism between ERα66 and ERα36. In fact, ERα66 appears as able to suppress the ERα36 activity. The loss of ERα66 expression related to an increase in ERα36 expression represents one of the mechanisms leading to the acquisition of resistance to antiestrogen therapy, e.g., by tamoxifen (28). This review highlights the effects of the ERα36 on several different cancer cells types.

## ERα36 in Different Types of Tumors

It is now known that estrogens, through their receptors, play an important role in the pathogenesis of many types of tumors. In particular, ERα36, has been demonstrated to be involved in tumor progression and growth, metastatic potential, resistance to treatment and poor prognosis (29). A high expression of this isoform has been described in some types of tumors, such as renal cell carcinoma, papillary thyroid carcinoma, laryngeal carcinoma, endometrial carcinoma, hepatocarcinoma, gastric cancer, neuronal tumors (neuroblastoma and glioblastoma), and breast cancer. However, whether ERα36 could play a role in other human cancers is still scarcely investigated and cannot be ruled out.

### Renal Cell Carcinoma

Dysregulated estrogen signaling contributes to the initiation and progression of renal cell carcinomas (30), but the mechanism has not been well established. Wang et al., in a retrospective study, hypothesized that ERα36 may be involved in tumor progression (31). In this study the authors described a different expression level and cell localization of ERα36 in malignant and benign renal tumor cells. In particular, a greater expression of ERα36 in malignant tumor cells compared to benign ones with a predominantly cytoplasmic localization in the latter has been shown. Furthermore, the high expression levels of ERα36 have been found related (i) to renal cell carcinoma necrosis, and (ii) to increased metastastatic aggressiveness. Further studies appear as mandatory in order to evaluate the potential role of ERα36 expression levels as prognostic markers in renal cell carcinoma and to differentiate benign from malignant tumors (31).

### Papillary Thyroid Carcinoma

Papillary thyroid cancer (PTC) represents about 80% of malignant thyroid tumors and is three times more common in women than in men suggesting a critical role of estrogen in its occurrence and development (32–34). Dai et al., analyzed the expression levels of ERα36, in association with the epidermal growth factor receptor (EGFR) and HER2, in PTCs, nodular hyperplasias and normal thyroid tissues (35). The results obtained highlight the existence of a significant correlation between the high levels of expression of ERα36 in PTC tissues and the progression and increase of tumor metastases. In particular, by correlating the expression levels of ERα36, EGFR and HER2 with the clinicopathological characteristics of PTC, high levels of these receptors significantly correlate with extrathyroidal extension and lymph node metastasis have been found. Conversely, there is no correlation between ERα36 expression and the histologic subtype, age, gender and tumor size of PTC patients. Hence, ERα36, in association with the expression of EGFR and HER2, could represent, if further validated, a possible marker of the tumor stage of PTC (35).

### Laryngeal Carcinoma

Significant differences between sexes have been described as concerns this carcinoma, with a male to female ratio of 11: 1 (36–38). This, suggests that sex hormones may be involved in the tumorigenesis of this form of cancer. Even though it is not uniformly accepted as a hormone-dependent tumor, laryngeal cancer expresses ERα36. By *in vitro* studies using laryngeal carcinoma epithelial cells (Hep2), the binding of ERα36 with E2 has been shown to induce a rapid activation of PKC and PLD, with an increase in the proliferative rate and in resistance to chemotherapy-induced apoptosis. In Hep2 cancer cell line, ERα36 is located in caveolae (sphingolipid and cholesterol rich plasma membrane microdomains) in association with caveolin-1 and, after E2 treatment, induces an upregulation of angiogenic and metastatic factors (39). By immunohistochemical analysis of human laryngeal tumors, an association between the amount of ERα36 and vascular endothelial-derived growth factor (VEGF) has been observed, supporting a role of ERα36 in vascularization and metastasis (39).

### Endometrial Carcinoma

Endometrial carcinoma is one of the most frequent gynecological malignancies in women (40, 41). Among the most common risk factors are polycystic ovary syndrome, obesity (42) and prolonged exposure to endogenous estrogens (43). Estrogens, in addition to increasing the risk of tumor onset, play a key role in the development and progression of endometrial carcinoma (40). Comparing endometrial cancer cells to normal cells, an increase in aromatase activity, the enzyme that converts androgens into estrogens, has been observed (44).

Interestingly, endometrial carcinoma cells (Hec1A) express ERα36 at plasma membrane and cytoplasm level (45). A positive correlation between ERα36 and EGFR expression levels has been observed in Hec1A cultured cells suggesting the involvement of ERα36 in the activation of the extracellular signaling linked to EGFR in endometrial carcinoma (45). Moreover, as observed in other tumors, ERα36 promotes the agonist activity of tamoxifen in endometrial cancer cells (23). In fact, both estrogen and tamoxifen are able to promote the activation of the MAPK/ERK and PI3K/AKT pathways through ERα36 (23). Furthermore, treatments with estrogens or tamoxifen induce the expression of the proto-oncogene c-Myc in Hec1A cells (23, 25). Therefore, ERα36 could be considered as a potential prognostic biomarker of endometrial carcinoma (46).

### Hepatocarcinoma

Hepatocellular carcinoma (HCC) is one of the most common malignancies and is the third cause of cancer-related death worldwide. The risk is increased in presence of chronic hepatitis and cirrhosis. The incidence of this carcinoma is 3 times higher in men than in women suggesting that sex hormones, estrogen in particular, could play a critical role in its development (47). In fact, some studies suggest that estrogens could exert a protective role in the development of HCC (48). Accordingly, the incidence of this carcinoma can be significantly lowered by estrogen treatment in post-menopausal women (49).

As concerns ERs, Miceli et al. (50) suggest that a switch from the expression of ERα66 to the expression of ERα36 could be associated with development and progression of human HCC. ERα36, poorly expressed in normal hepatocytes, is instead well expressed by hepatocarcinoma cells and is localized at the plasma membrane as well as in the cytoplasm, supporting the idea that it could be involved in HCC development and/or progression (50). Other studies highlight more complex role of ERα36. Its expression seems in fact to be higher in primary HCC in comparison with secondary HCC and it appears as inversely correlated to ERα66 expression (21). Hence, these authors hypothesize that the expression level of ERα36 might be considered a useful tool to differentiate the primary and secondary HCC. Further insights also derive from studies carried out with epigallocatechin-3-gallate (EGCG), a natural product that exerts its anti-cancer in HCC by inhibiting ERα36 (51). Hence, ERα36 appears to play a role in the pathogenesis of HCC, but further studies are needed to better understand the exact role of the different ER isoforms in this cancer.

### Gastric Cancer

To date, the molecular and cellular mechanisms involved in the development of gastric cancer are still to be elucidated. Some studies suggest a protective role for estrogen in the development of gastric cancer (52–54). In fact, the incidence of this cancer is higher in men than in women before menopause and tends to increase in women after menopause (53). Other data highlight an involvement of estrogen in the tumorigenesis of gastric cancer (55). In this regard, ERα36 appears to be highly expressed in gastric cancer cell lines with a mainly cytoplasmic and/or surface localization. In addition, ERα36 expression is significantly associated with lymph node metastasis but not with the other clinicopathological features of gastric adenocarcinoma. Therefore, the increase of ERα36 expression in gastric adenocarcinoma and its association with metastasis could suggest that the evaluation of ERα36 level could represent a prognostic biomarker for gastric cancer progression (56). However, opposite results have been reported by another study conducted by Wang et al. These authors reported a lower expression of ERα36 in tumor tissues than in normal tissues and an expression level of ERα36 negatively correlated with the tumor size and the number of metastases (57). On these bases, the need of further studies aimed at clarifying the effective role of ERα36 in gastric cancer onset and progression appears well evident.

### Neuroblastoma

Neuroblastoma is a very aggressive solid tumor that occurs most frequently in children after leukemia and brain cancer. It is an embryonic tumor that originates from the sympathetic nervous system (58). In recent years, several lines of evidence have demonstrated that ERα can contribute to neuroblastoma tumorigenesis. In particular, the expression of ERα appears to be related to neuronal differentiation and to the survival rate of patients with neuroblastoma (59, 60). *In vitro* studies in human neuroblastoma SH-SY5Y cells, the knocking down of the ERα36 gene with the specific siRNA lead to a reduction of cell proliferation and an increase in apoptotic susceptibility. In particular, the silencing of ERα36 seems to be associated to a reduction in protein phosphatase 2A (PP2A) activity (of importance in cellular homeostasis and tumor suppression) and an increase in phosphorylation of the tau protein (of importance in cytoskeletal integrity and function). In addition, ERα36 gene silencing has been shown to reduce the expression of Cyclin Dl, the proliferating cell nuclear antigen (PCNA) and B cell lymphoma-2 (Bcl-2) antiapoptotic protein while increasing the expression of proapoptotic protein Bax. Furthermore, the regulation of some pathways such as MAPK/ERK and PI3K/AKT has been shown to be dependent on the interaction between Caveolin-1 and ERα36, i.e., on lipid raft function (61).

### Glioblastoma

Glioblastoma (GBM) is a highly aggressive and highly invasive primary brain tumor. Patients with GBM have a poor prognosis with an average survival of approximately 1 year (62). Although several studies have shown that adjuvant treatment with tamoxifen could be capable of sensitizing glioblastoma cells to radiation therapy also inhibiting their proliferation, this approach cannot be used for all types of glioblastoma. In addition, long-term use of tamoxifen can lead to the induction of tamoxifen resistance (63, 64). Therefore, several studies have been conducted to understand the molecular mechanism of tamoxifen resistance and to improve the quality of life of patients with glioblastoma (65). In this regard, an involvement of ERα36, that is highly expressed in GBM, has been suggested as pivotal in the induction of resistance to tamoxifen treatment. In fact, Qu et al. observed that high levels of ERα36 could block the tamoxifen-mediated cell growth inhibition and induce autophagy by hindering the AKT/mTOR signaling pathway. However, the effect of autophagy on tumor cell viability is still to be elucidated. Indeed, it seems that in conditions of nutrient and oxygen deficiency, when the tumor size increased, autophagy could promote the survival of cancer cells. Accordingly, *in vitro* studies on glioblastoma cells treated with tamoxifen showed a significant increase in ERα36 expression level accompanied by an increased cytoprotection by autophagy. These results provide new insights into the mechanism underlying the antiproliferative, cytostatic, properties of tamoxifen and the involvement of ERα36 in resistance to tamoxifen treatment (65) also suggesting the contribution of autophagy pathway to the development and progression of glioblastoma (62).

### Breast Cancer

This is the most common form of cancer among women. About 70% of breast cancers express ERα66, which is involved in the transcriptional regulation of estrogen-sensitive genes (66–68). Among the main therapeutic treatments used, there are anti-estrogens, which tend to block the molecular pathogenic pathways mediated by ERα66. Unfortunately, many patients develop *de novo* or acquired resistance to these therapeutic agents, which is associated with the onset of metastasis and poor prognosis (69–71). Recently, ERα36 has been found expressed in ER-positive and ER-negative breast cancer cells (20, 24, 72). ERα36 expression levels have been associated with some clinicopathological features of breast cancer (tumor size, clinical stage, histological grade, lymph node metastasis) (28). Triple negative breast cancers (TNBC), i.e., lacking of ERα66, progesterone receptor (PR) and epidermal growth factor receptor (EGFR), are very aggressive tumors with high recurrence, elevated mortality rates and limited therapeutic options. Maczis et al., observed that ERα36 is expressed in TNBC and is involved in E2-induced activation of sphingosine kinase 1 (SphK1) and for the production of shingosine-1-phosphate (S1P), which has a role in tumor growth, progression, transformation and metastasis (73). Interestingly, tamoxifen could act as an agonist of ERα36 and induce proliferation, invasion and metastasis in breast cancer cells. In support of these data, Wang et al. observed that ERα36, through the tamoxifen agonist activity, can be able to increase the expression of aldehyde dehydrogenase 1A1 (ALDH1A1), a molecule involved in cancer stem cell maintenance and metastasis. In particular, treatment with tamoxifen induces the nuclear translocation of the ERα36 receptor, which directly regulates the transcription of ALDH1A1, suggesting a genomic-type mechanism of action in stem/progenitor cells of ER-positive breast cancers (29, 74).

Previous studies have shown that there are several molecular mechanisms and/or pathways involved in treatment resistance, including EGFR and HER2 (69, 75). Recently, the existence of a positive correlation between the levels of expression of ERα36 and EGFR/HER2 has been suggested to be involved in the mechanism of resistance to tamoxifen and in the increased proliferative capacity of breast cancer (20, 27, 69, 75–77). The activation of the SphK1/S1P/S1PR axis mediated by E2 through ERα36 could be one of the mechanisms that lead to the development of *de novo* and acquired resistance to anti-estrogenic therapy of breast cancer (73). In addition, a positive correlation between I-kappa-B-kinase-epsilon IKKε (an oncogene, member of the IKK family) and ERα36 has been observed (78). In particular, IKKε interacts with ERα-36 and increases its expression. IKKε seems to promote the mitogenic signaling of estrogens through ERα36 with the consequent induction of proliferation in ER-negative breast cancer cells (78). To note, in ER-negative cancer cells, ERα36 can inhibit and/or activate the EGFR/signal transducers and activators of transcription 5 (STAT5) pathway through the regulatory function of Src. *In vitro* studies on ER-negative cells show how low concentrations the tamoxifen are able to induce the activation of MAPK/ERK pathway whereas at higher concentrations the signal is turned off. This could be explained by the fact that different concentrations of anti-estrogens could lead to different conformations and/or functions of ERα36 (79). Overall, even though several molecular mechanisms are involved in the occurrence of anti-estrogenic resistance, ERα36 seems to contribute to these mechanisms also playing a role in the maintenance of stem/progenitor cells of breast cancer.

Recently, the presence of serum autoantibodies reacting with ERα (anti-ERα Abs) in a large percentage of patients with breast cancer has been shown (80, 81). *In vitro* studies with anti-ERα Abs purified from sera of patients by affinity with the recombinant ERα66, allowed us to observe that they bind to and activate ERα expressed at membrane level, located within the lipid rafts triggering rapid MAP kinase activation and inducing tumor cell proliferation (80). Moreover, anti-ERα Abs are also able to interfere with the efficacy of tamoxifen treatment suggesting that they can react and activate also the ERα36 isoform (81). However, in contrast, anti-ERα Abs showed no reactivity with the ERα66-negative, ERα36 positive MDA-MB-231 cells, indicating that ERα36 epitopes could be not accessible to antibodies, perhaps for conformational modification in these tumor cells. To note, treatment with simvastatin, removing or lowering cellular cholesterol, an integral component of lipid rafts (82, 83), inhibits anti-ERα Abs effect on proliferation and cell cycle progression (81).

Finally, even though it has been observed that cell surface ER could activate intracellular pathways, the precise mechanisms are still unknown. An interaction with receptor tyrosine kinases and/or with G protein-coupled receptors could be involved in downstream signaling pathways (e.g., phosphatidylinositol-3-kinase, Akt/protein kinase B and the mitogen-activated protein kinase cascade). Moreover, we also cannot exclude a cross-reaction of these autoantibodies with GPR30 leading to a rapid non-genomic signals. In all cases, the identification of autoantibodies reacting with mERα could be considered as a potential prognostic and predictive tool for breast cancer.

## Conclusions

Summarazing, it can be hypothesized that ERα36 could play an important role in estrogen signaling during the development and progression of several forms of cancer. However, to date, some open questions remain unanswered.

(i) The first one regards the potential role of ERα36 in further forms of human cancers, in particular, those presenting significant sex/gender differences in terms of incidence or progression such as melanoma and colon cancer.
(ii) The second open question concerns the potential therapeutic usefulness of statins. These drugs, impairing ERα36 function in patients with estrogen-dependent cancers, could exert some beneficial effect.

Further studies are thus mandatory in order to clarify both these aspects but, also, to better evaluate the role of ERα36 in the clinical practice as prognostic biomarker and/or as therapeutic target leading to the reduction of tumor growth and progression and/or reducing the occurrence of anti-estrogenic therapy resistance.

## Author Contributions

MP study conception and design, manuscript drafting. EO study conception and design, critical revision. MD study conception and design, manuscript drafting, critical revision. All authors contributed to the article and approved the submitted version.

## Conflict of Interest

The authors declare that the research was conducted in the absence of any commercial or financial relationships that could be construed as a potential conflict of interest.
